# Supplementary material for: Accurate Detection of Salmonella Based on Microfluidic Chip to Avoid Aerosol Contamination
Source: Foods. 2022 Dec 1;11(23):3887. doi: 10.3390/foods11233887 (PMC9740996; doi:10.3390/foods11233887)
Supplement: Supplementary file 1 [file foods-11-03887-s001.zip › foods-1926119-supplementary.pdf]

Supplementary Information

Accurate Detection of *Salmonella* Based on Microfluidic Chip to Avoid Aerosol Contamination

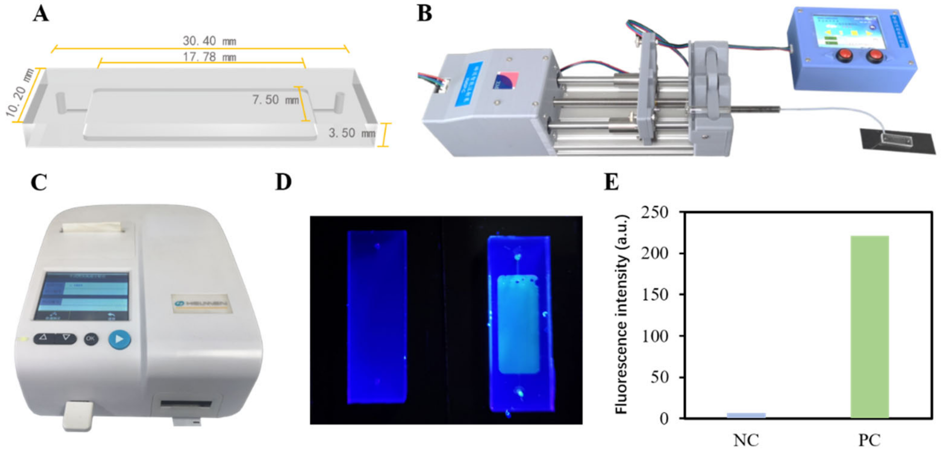

Figure S1. Devices and the effect diagrams. (**A**) PDMS chip model marked with actual size. (**B**) Smart syringe pumps and microinjectors for injection. (**C**) Fluorescence immunochromatographic reader. (**D**) Results of naked eyes observation of *Salmonella* detection on PDMS chip, negative and positive results respectively. (**E**) Negative and positive results read by fluorescent immunochromatographic reader.

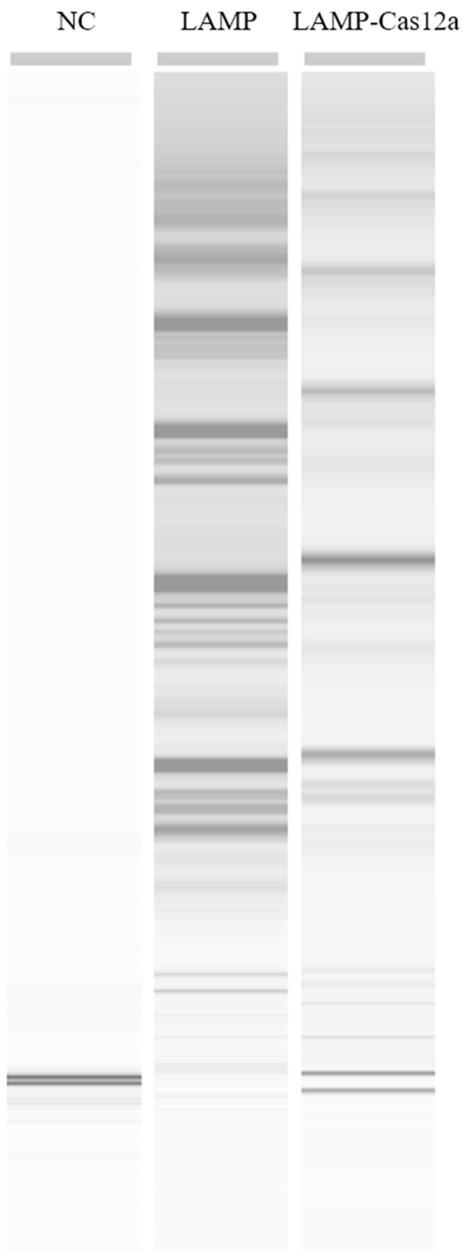

Figure S2. Electropherogram of negative control, LAMP products and LAMP products cleaved by Cas12a.

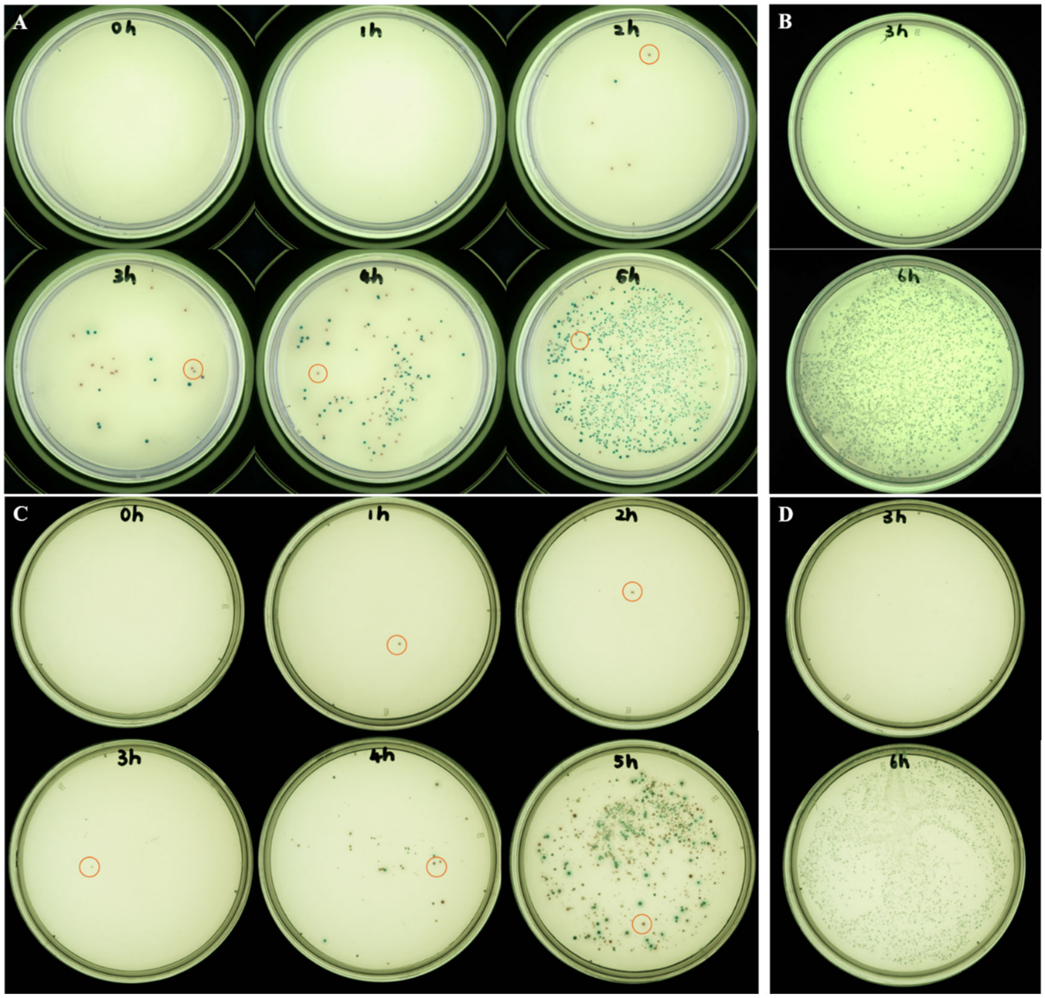

Figure S3. Culture results of bacteria solution collected from (**A**) spiked salmon sample, (**B**) un-spiked salmon sample, (**C**) spiked chicken sample and (**D**) un-spiked chicken sample at different times on the *Salmonella* chromogenic medium, in the matrix test. Purple colonies circled were *Salmonella* anthropogenically added, and blue colonies were *Escherichia coli* from sample.
